# Supplementary material for: Are carbon emissions trading and green financial instruments synergistic? -Comprehensive quantitative research based on content analysis
Source: PLoS One. 2024 Mar 7;19(3):e0298601. doi: 10.1371/journal.pone.0298601 (PMC10919601; doi:10.1371/journal.pone.0298601)
Supplement: S1 Data — (ZIP) [file pone.0298601.s001.zip › Data/地级市Z_F工作报告.zip!/─■╧─/╣╠╘¡╩╨/└┤╫╘╣1⁄2╓┌║┼í╛┬φ┐╦╩2╛▌═°í┐_2017.docx]

各位代表：

　　我代表市人民政府向大会报告工作，请予审议，并请市政协委员和列席人员提出意见。

　　本届政府工作

　　2012年12月本届政府产生以来，深入贯彻党中央、国务院系列决策部署，在自治区党委、政府和市委的坚强领导下，在市人大及其常委会的法律监督和市政协的民主监督与支持下，我们认真践行真诚服务人民的宗旨和誓拔穷根的诺言，统筹做好稳增长、促改革、调结构、惠民生、保稳定工作。地区生产总值、固定资产投资、地方财政预算收入和支出、农村居民人均可支配收入、社会消费品零售总额等主要经济指标增幅连续四年居全区五市前列;我市生态建设、民族团结、扶贫开发在全国创造了经验，生态旅游、绿色农业、文化建设在西部形成了优势，“三苦”作风、劳动力素质提升和“两个带头人”工程、“六分离”公共资源交易方式、“老饭桌”模式在全区打造了亮点。奠定了打赢脱贫攻坚战、全面建成小康社会的坚实基础。

　　一、勇于改革创新，固原迈出新步伐。强力改革行政体制，构建开放新格局，拓展发展新空间。一是思想解放深化。认真学习习近平总书记系列重要讲话精神和治国理政的新理念新思想新战略，不断增强贯彻落实党的路线方针政策的自觉性和坚定性。组织干部走出去学经验、找差距，邀请专家学者来固把脉问诊，促进了全市上下进一步更新发展观念，增强发展意识，公职人员依法行政、谋事成事、服务群众、服务企业的能力逐步提高。二是发展活力增强。推进“放管服”改革，在全区率先公布市级权责清单，发布项目准入负面清单，精简归并市级行政审批事项231项，减少行政事业性收费项目330项，减幅分别为61.3%和89%;在全区率先实行企业注册“五证合一”、个体工商户“两证合一”、食品药品许可“六证合一”、房产交易“两证一税”联办服务，全面落实“营改增”政策，助推非公经济发展，新增市场主体1.7万户，年均增长10.3%。加大财政和投融资体制改革，采取PPP模式融资建设海绵城市等项目，组建了九龙、交通、旅游、农投4家集团公司，创建了资源优化组合、资本市场化运作的新路子。三是开放通道打开。彭青、东毛、固西、李庄至泾河源高速和西毛一级公路等建成通车，实现县县通高速，新(改)建普通国省道路和农村公路3685公里;西吉至会宁、泾源至华亭高速、彭阳至镇原二级公路即将开工建设;新增了到重庆、上海、天津航线，加快与发达地区和周边互联互通。启动“智慧固原”“宽带中国”示范城市建设，“扶贫云”“旅游云”“政务云”落地运行，贫困村光纤宽带全覆盖。四是合作领域拓展。主动融入“一带一路”和宁夏内陆开放型经济试验区建设，加强与港澳台、沿海发达省区的交流合作;全面加强与陕甘宁蒙青毗邻地区联系，落实与平凉、庆阳等周边城市合作协议，联合争取政策项目支持区域发展。成功举办了第六届中国宁夏六盘山冷凉蔬菜节、第十届牛业发展大会等一系列重大节会，招商引资累计到位资金628.5亿元，年均增长26%。

　　二、聚焦精准扶贫，固原跃上新台阶。坚持精准脱贫与整村推进相结合，尽力补齐全面小康的突出短板。一是脱贫攻坚精准有效。认真落实“六个精准”“五个一批”要求，实施“1+21+X”精准扶贫规划，四年共脱贫销号贫困村339个，搬迁安置生态移民3.4万户14.4万人，贫困人口由44.8万减少到17.1万，贫困发生率由35.2%下降到15.4%。习近平总书记在中央扶贫开发工作会议上和来宁视察时，对我市扶贫开发工作给予了充分肯定。国家部委、闽宁协作、部门单位、企业商会、慈善组织、军警部队参与的“1+6”社会帮扶体系逐步完善，落实帮扶资金13.5亿元，“闽宁模式”成为全国东西扶贫协作的典范。“两个带头人”工程培育脱贫中坚力量，选派贫困村第一书记735名，培育农村致富带头人3506名，脱贫工作力量不断壮大，引领群众致富奔小康。劳动力素质提升工程促进农民稳定增收，围绕增收产业、致富项目，实施10万劳动力技能培训拿证工程，已培训6.79万人，拿到国家职业资格证书4.37万人，贫困群众“造血”功能明显增强，在传统务工机会减少的前提下，工资性收入支撑农民持续增收。金融扶贫工程助力产业加速富民，成立融资担保公司7家，建立各类担保基金10.97亿元、风险补偿基金1.2亿元，发放扶贫贷款69.4亿元，有效解决了贫困户发展资金短缺难题，金融扶贫创造了全区扶贫开发新模式。二是优势产业提质增效。认真落实产业结构调整优化升级实施方案和加快五河流域发展若干意见，安排3.1亿元产业扶持资金，加快“3+X”产业转型升级，产业结构由2012年的23.7∶26.9∶49.4调整为2016年的20∶28∶52。特色农业优势彰显，建成宁夏六盘山百万头肉牛养殖基地、西北重要的马铃薯脱毒种薯繁育基地和苗木种植基地;冷凉蔬菜全部通过无公害农产品产地认证，畅销国内大中城市和港澳地区。我市被授予中国“冷凉蔬菜之乡”“马铃薯种薯之乡”和“六盘山黄牛之乡”;六盘山农产品、六盘山苗木荣获中国驰名商标，六盘山珍食用菌等27件商标获宁夏著名商标，泾源黄牛肉、彭阳辣椒等获农业部地理标志登记认证。严守耕地红线，耕地稳定在503.8万亩，粮食总产稳定在80万吨左右。农业增加值年均增长5.1%。新型工业稳步提升，建成“一区五园”发展载体，整合提升市区产业园区，集聚效应不断增强。培育引进工业企业85家，累计达到815家，规上企业从23家增加到56家，共吸纳就业3.3万人。金昱元100万吨真空制盐、40万吨电石等项目投产，王洼煤矿千万吨级矿区改扩建等项目基本建成，六盘山750KV变电站建成投运。工业增加值年均增长12.8%。现代服务业势头强劲，全域旅游实现新突破，实施了六盘山国际旅游休闲度假区、泾源旅游服务带等项目;须弥山石窟、火石寨国家地质森林公园晋升为国家4A级景区，西吉龙王坝、隆德红崖、彭阳杨坪入选“中国最美休闲乡村”，六盘山红军长征纪念馆和将台堡红军会师纪念碑入选全国“我心中的长征纪念地”。确定和大力宣传“天高云淡六盘山”品牌，旅游人数和收入分别是2012年的2倍和2.3倍。五县(区)全部进入国家农村电子商务示范县行列，建成5个县级电商服务中心和405个农村电商服务站。扶持传统服务业改造升级，新华百货等大型商业和西港航空酒店等高级酒店建成运营。金融保险业恢复性增长，加强政银企对接，引进石嘴山银行、津汇村镇银行等金融机构4家，全市金融机构存贷比72.97%，较2012年上升17.89个百分点。服务业增加值年均增长9%。三是社会保障扩面提效。每年安排2000万元扶持“双创”孵化基地建设，建成创业园区(孵化基地)49个，六盘山创客汇被授予国家小型微型企业创业创新示范基地;累计发放创业担保贷款14.55亿元，创业带动就业和新增城镇就业8万多人。每年稳定转移农村劳动力30万人，劳务收入占农民收入43%左右。改造农村危房危窑10.1万户，基本消除农村危房危窑;建成保障性住房1.9万套。建立被征地农民养老保险“先保后征”机制，城乡居民基本养老保险全覆盖。推进农村最低生活保障制度与扶贫开发政策有效衔接，实现“两线合一”，城乡低保人均补助标准分别提高12%和26.8%;社会救助体系更加完善，群众获得感进一步增强。

　　三、增进民生福祉，固原取得新进步。财政支出80%以上用于民生事业，为民承诺的70件实事全部办结。宁夏中南部城乡饮水安全水源及连通工程建成通水，圆了百万城乡居民的安全饮水梦想。一是教育文化普惠百姓。投资118亿元，新改扩建校舍、运动场等7443万平方米，上学条件显著改善。原州区、隆德县、泾源县、彭阳县义务教育均衡发展工作通过自治区验收，农村义务教育学生营养改善计划全面落实;在全区率先实施“9+3”免费中等职业教育，中职示范校建设迈上新台阶;全市学前三年毛入园率达到72.1%，较2012年提高37.5个百分点;高考上线率连年高出全区平均水平。市图书馆、文化馆、丝路文化展览馆主体基本建成;广播电视户户通、农民体育健身工程和农家书屋村级全覆盖，文学固原、农村文化大院成为特色品牌。成功举办了两届全国群众登山健身大会暨宁夏六盘山登山节等活动，全民健身中心免费开放，全民健身运动广泛开展。二是医疗卫生改革便民。医药卫生体制改革稳步推进，在全市推广“先诊疗后付费”“先住院后付费”服务模式，在全区率先建立市级统筹城乡医疗保险、实施人人享有基本医疗服务和城乡居民大病医疗保险政策，居民实际报销支付比例达到63.3%，群众就医负担明显减轻。实施市、县、乡医院新改扩建项目59个，标准化村卫生室全覆盖。计划生育工作扎实有效，人口自然增长率控制在10‰以内。三是平安成果人人共享。深入践行社会主义核心价值观，弘扬优良传统，凡人善举层出不穷，向上向善蔚然成风，诚信感恩扎根民心。完成“六五”普法，启动“七五”普法。积极推进“平安固原”建设，创建基层矛盾纠纷排查化解“134”机制，社会治安立体化防控体系建设不断深化，群众安全感连续五年居全区五市第一。食品药品安全监管不断加强，安全生产形势持续稳定。扎实开展民族团结进步创建活动，“三个离不开”思想更加深入人心;宗教工作“5+6+1”制度全面落实，宗教事务法治化、规范化管理水平进一步提升，全市民族团结、宗教和顺、社会和谐的局面进一步巩固。四是作风建设从严从实。扎实开展党的群众路线教育实践活动、“三严三实”专题教育和“两学一做”学习教育，严格执行中央“八项规定”，干部作风明显转变，政府效能明显提升，自身建设明显加强。2013年、2014年获全区五市效能目标管理考核一等奖。健全依法决策机制，建立完善政府法律顾问、政府性资金审计管理、政务公开、行政效能问责等制度;全面落实市委决定事项，提高市县乡代表、委员履职经费保障标准，149件区市人大代表议案、建议，732件政协委员提案全部办结。认真落实党风廉政建设责任制，强化审计监督和行政监察，着力纠正损害群众利益的问题和行业不正之风，政风行风评议群众满意度逐年上升。社会信用体系建设取得新成果，城市信用排名居宁夏第一、全国地级市第53名(除首府城市)。争取国家和自治区各类补助资金620亿元、亿元以上项目306个。截至目前，全市债务余额88.88亿元，负债率34%，债务率39%，通过负债配套落实国家、自治区项目资金，形成土地、市政设施、民生工程等356亿元优质资产。国防动员深入推进。统计、人防、地震、气象、档案、地方志、老龄、妇女儿童、外事侨务等工作取得新的进步。

　　四、坚持生态优先，固原展现新画卷。认真落实加快固原建设实施意见，天蓝、地绿、水清、宜居、宜游的新固原建设进程加快。一是生态更靓丽。实施六盘山重点生态功能区、新一轮退耕还林、移民迁出区生态修复等重点工程，新增营造林192.4万亩，森林覆盖率达到22.8%;完成104处农村安全饮水、84座中小型水库提升改造、11处抗旱应急水源建设等工程，高效节水灌溉面积达到45万亩;新修旱作基本农田36万亩，治理小流域1600平方公里，被确定为全国生态文明示范工程试点市和生态保护与建设示范区。积极开展大气、水、土壤污染防治行动，认真整改中央环保督察组反馈的问题，淘汰关停燃煤锅炉及茶浴炉126台，市区和各县城实现集中供热，空气质量优良天数保持在88%以上;建立渝河、葫芦河跨界联防联控协作机制，共同推进跨境流域水体治理，渝河、葫芦河水质恶化势头得到遏制，渝河出境断面水质达到Ⅳ类。关闭万吨以下马铃薯加工企业175家，市县城市污水处理厂和垃圾填埋场、农村环境连片整治项目全覆盖，农田残膜回收率达到85%以上。二是城乡更美丽。围绕“1411”城镇体系，推进国家新型城镇化综合试点城市和海绵城市建设，完善新区功能，开建西南新区，实施旧城改造，市区建成区从36.62平方公里扩大到44.1平方公里。集中实施了一批学校医院、市政设施、房产商贸项目，打通市区首条环城路和南北两个高速进出口，建成城市规划展示馆、博物馆应急避难广场等标志性工程和人民公园、原州古雁岭、东岳山、西吉北山、隆德龟山、泾源卧龙山、彭阳茹河等7个市民休闲森林公园，城镇建成区绿化覆盖率达到36.69%。实施了西吉棚户区改造、隆德渝河县城段生态景观、泾源德通商务广场、彭阳悦龙山新区开发等县城扩容提质工程，打造了三营、兴隆、联财、六盘山、王洼等8个中心镇和驼巷、单南、红崖、兴盛、韩堡等36个美丽村庄。坚持建管并举，开展“五城联创”活动，成功创建自治区卫生城市和双拥模范城市;三营镇被列为全国建制镇示范点，泾河源镇入围全国首批特色小镇。全市常住人口城镇化率从34.85%提高到46.08%。

　　各位代表，即将过去的2016年，在我市发展史上具有特殊意义。习近平总书记亲临我市视察指导，李克强总理深入一线考察慰问，更加坚定了我们打赢脱贫攻坚战、全面建成小康社会的信心和决心!全市上下以贯彻习近平总书记来宁视察重要讲话精神为动力，全面落实市委三届七次全会总体要求，推进“六个基础”和“六大工程”建设，主要指标除居民收入增速外，均完成了市三届人大四次会议确定的目标任务，实现了“十三五”规划的良好开局。预计2016年，全市完成地区生产总值240亿元，增长8%以上，年均增长9.4%;地方公共财政一般预算收入13.89亿元，可比增长10%，可比年均增长13.55%;全社会固定资产投资391亿元，增长13%，年均增长22.1%;城镇居民人均可支配收入22941元，增长8.5%，年均增长9.1%;农村居民人均可支配收入7646元，增长9.2%，年均增长11.3%;各项约束性指标均完成自治区下达的任务。一是脱贫任务全面完成。整合投入各类资金22亿元，实施富民产业、基础设施、危房危窑改造等精准扶贫工程包，启动“十三五”易地扶贫搬迁，打出了脱贫攻坚“组合拳”，预计销号贫困村150个、减贫9.6万人。脱贫成效受到国务院扶贫办委托的第三方评估组认可。二是“3+X”产业优势增强。草畜产业引进万家灯火、天山牧业等龙头企业带动发展，新增肉牛3.1万头，其中安格斯肉牛1.2万头;林业和林下经济突出生态经济效益，中药材、油用牡丹等种植增长12.5%，中蜂养殖增长24.2%;全域旅游启动了张易至隆德、羊坊至中庄水库等4条环线建设，乡村旅游和“农家乐”旅游发展迅速，带动更多的农民进入了旅游市场。全市接待游客量和旅游社会总收入分别增长23%和21%。三是旧城改造深得民心。开工建设海绵城市部分项目。全市棚户区改造完成征地拆迁1.5万户，兑付补偿资金44.5亿元;市区博物馆、西湖路等6个片区开工建设，实现了群众拥护、住房改善、房地产去库存、城市变美的“多赢”局面。四是民生保障持续给力。新改建乡村幼儿园39所，资助家庭困难学生4.3万名;完成市医院迁建和544所村卫生室温暖工程;为5.6万农户安装了太阳能热水器;新建特色农村文化大院84个，累计达到128个;购置新能源公交车30辆，发展成果更多地惠及全市人民。五是重点改革落地见效。成立市行政审批服务局，行使相对集中行政许可权。完成了土地确权颁证，推行土地股份制、土地承包经营权和林权抵押贷款试点等农村改革;小型水利工程管理体制及农业水价综合改革走在全区前列。理顺了城市、旅游、市区工业园区管理体制。人才工作机制不断完善。完成市县公车改革。城市公立医院综合改革全面推开，取消药品加成，政府办医的责任进一步强化。

　　回首本届政府工作，特别是通过谋划“十三五”大调研活动，我们找准了一条以脱贫攻坚为统领、推动加快发展、跨越发展的新路子，建成了一批强基础、利长远的重大项目，办妥了一批民生所需、民心所盼的实事好事，突破了一批制约发展的体制机制障碍，全市经济发展更加持续、改革之势更加强劲、民生福祉更加丰厚、生态环境更加优美、社会大局更加和谐。在此，我代表市人民政府，向各位代表和委员，向全市各条战线上的劳动者、各民主党派、工商联、人民团体、社会各界和中央、自治区驻固单位、人民解放军、武警官兵及公安干警，向所有关心支持固原建设和发展的朋友们，表示崇高的敬意和衷心的感谢!

　　各位代表，对标全面建成小康社会，前进的路上仍存在许多困难和挑战。一是跨越发展的基础不强。发展不足是我市最大的市情，经济总量小，财政自给能力弱;产业层次低、链条短，产城融合不紧密、就业渠道单一，促进居民持续增收困难大;招大商、引人才难，好项目储备少，交通、水利、产业、信息瓶颈还未根本破解;生态环境依然脆弱，五河流域水污染防治任务重，PM10不降反升。二是精准脱贫的内生动力不足。劳动力技能培训与产业发展、市场需求对接不紧密;金融扶贫覆盖面窄，扶贫资金整合机制不健全;鼓励创新创业的政策激励不强，新的经济增长点培育缓慢;教育、医疗、就业等公共服务水平与群众期望还有差距。三是社会治理深度不够。安全隐患整治任务重，治安防控技术落后、盲点多;城区违建行为屡禁不止，一些群众表达诉求信访不信法;婚丧嫁娶高额礼金风气盛行，信教群众宗教负担较重;贪污、挪用扶贫资金、涉农资金等群众身边的腐败问题时有发生。四是干部创新担当精神不佳。面对经济新常态、脱贫新任务和小康新要求，一些干部思想因循守旧，谋事眼界不宽，干事方法落后，等靠要思想仍然存在，争项目、争资金的劲头不足;有的干部缺少担当精神和责任意识，工作效率和服务质量不高;个别部门单位还存在懒政怠政不作为现象，等等。我们必须坚持问题导向，补齐突出短板，不负人民重托。

　　新一届政府工作

　　今后五年，既是固原脱贫攻坚、全面建成小康社会，实现第一个百年目标的决胜期，也是我市补齐交通、水利、产业、人才等短板的机遇期。纵观大势，机遇大于挑战，国家实施“一带一路”、供给侧结构性改革、脱贫攻坚等重大战略机遇叠加;自治区党委、政府对固原的特殊支持还将持续加大;全市群众盼富求强，干部勤勉敬业，蕴藏着巨大的创新创业力量。我们坚信，只要坚持市第四次党代会确定的发展蓝图不动摇，大力发扬“不到长城非好汉”的宁夏精神，不忘初心，继续前进，就一定能够走好新的长征路，翻越脱贫攻坚这座大山，踏上全面小康的伟大征程。

　　今后五年的发展思路：高举中国特色社会主义伟大旗帜，以邓小平理论、“三个代表”重要思想、科学发展观为指导，深入学习贯彻习近平总书记系列重要讲话精神和治国理政新理念新思想新战略，围绕“五位一体”总体布局和“四个全面”战略布局，牢固树立创新、协调、绿色、开放、共享的发展理念，以脱贫攻坚为统领，以保障和改善民生为根本，以改革开放和科技创新为动力，以提高人的素质为核心，夯实交通、水利、教育、生态、城镇化、信息化“六个基础”，实施劳动力素质提升、两个带头人、产业提质增效、民生改善、旧城改造、金融扶贫“六大工程”，坚持项目带动，加强依法治理，维护社会稳定，巩固民族团结，全面从严治党，加快建设固原，为打赢脱贫攻坚战、全面建成小康社会而奋斗。

　　发展目标：经济发展、居民收入增速高于全区，生态建设、社会治理水平走在全区前列，民生发展、信息化建设与全区同步。到2021年，地区生产总值年均增长8%，全社会固定资产投资年均增长12%，地方公共财政一般预算收入年均增长9%，城乡居民人均可支配收入年均分别增长9%和10%。

　　各位代表，责任考验担当，实干成就伟业。我们必须把落实五大发展理念贯穿始终，必须把打赢脱贫攻坚战作为政府履职尽责的头号工程，必须把重实干、求实效、不出事作为干事创业的刚性要求，以不破楼兰终不还的气概，落实好以下五大重点任务。

　　一、坚持创新，培育全面小康核心动能。认真落实习近平总书记关于“越是欠发达地区，越需要实施创新驱动发展战略”的指示精神，把创新作为最关键的动力和结构调整、机制优化、方式转变的重要引擎，着力提高发展质量和效益。一是搭建科技创新平台。出台加快实施创新驱动发展战略意见，加强与大专院校、科研院所、企业合作，建立科技创新机制，到2021年，全市研发投入强度达到GDP的2%以上。市区建成国家技术创新中心1个，四县各建成自治区级技术创新中心1个，以此带动特色产业优良品种培育、高新技术应用、机械化推广等方面换挡升级。支持龙头企业建立研发中心和重点实验室，开发新产品、研发新工艺，建成国家级农业科技园区。构建集风景直播、语音导游、网上预订等功能为一体的智慧旅游服务体系。二是推进制度机制创新。以观念创新为先导，改革不利于发展和要素激活的体制障碍，让制度创新与科技创新双轮驱动发展。建立整合财力、人力集中办大事、建关键项目的发展机制，市上重点抓好规划指导、项目谋划、政策扶持、督查落实等方面的工作，县(区)突出抓好项目政策落地、责任措施落实等方面的工作;建立争项目、争政策、争资金，招好商、招人才、招技术，服务企业、服务人民、服务社会的工作机制(三争三招三服务);建立产业+龙头+财政+金融+市场的脱贫致富机制，让穷人跟着能人走、能人跟着产业走、产业跟着市场走;建立人才培养引进和激励机制，市财政每年安排3000万元推动人才工作，加快培养引进教育、医疗、金融、旅游、工业、信息等方面的创新创业人才，激发现有人才释放活力、开拓创新、激情干事，鼓励科技人员领办创办实体和开展技术有偿服务。三是创新产业发展模式。加快构建“市场牵龙头、龙头带基地、基地连农户”的农业模式，“全景、全业、全时、全民”的旅游模式，“市县共建、集群发展、低成本运行”的工业模式，形成业态对标市场、模式符合需求、产业+互联网、一二三产融合发展的现代产业体系。建设现代生态农业，围绕“一特三高”标准，构建产业、生产、经营体系，推动五河流域特色农业发展。到2021年，全市肉牛饲养量达到122万头，其中安格斯肉牛10万头;种植马铃薯170万亩、冷凉蔬菜65万亩、中药材20万亩，建成全国安格斯肉牛繁育基地、马铃薯种薯生产基地、冷凉蔬菜产业基地和六盘山道地中药材产业基地。把六盘山生态农产品打造成全国驰名品牌。建设全域旅游城市，引进大型企业开发旅游资源，建成具有国内一流标准的1条旅游环线和6条精品线路，改造提升森林公园、须弥山、火石寨、长征景区4个重点景区，打造沙塘、古城等19个特色旅游小镇，建成龙王坝、冶家村等45个旅游扶贫示范村，黄铎堡、杨家店等6个自驾营地和彭阳梯田公园。打响“天高云淡六盘山”生态旅游品牌，带动更多群众端上旅游“金饭碗”。到2021年，全市接待游客1200万人次，旅游社会总收入100亿元以上。建设新型工业园区，坚持生态、环保、节水优先的发展理念，重点发展农副产品加工和劳动密集型产业，选择发展能源新材料产业。按照“一区五园”布局，整合资源优势，市县共建共享，促进工业园区化、集群化、差别化发展，实现工业增加值翻番。依托盐矿资源和金昱元等企业，建设重要的国家级盐化工产业基地。到2021年，固原经济技术开发区升格为国家级开发区。

　　二、注重协调，建成宜居宜业美丽家园。坚持一手抓精准扶贫、一手抓区域发展，构建“山川共济、城乡协调、产城融合、生态文明”的发展格局，实现农业现代化、工业集群化、城镇新型化、社会信息化同步推进，协调发展。一是强化规划引领。高标准完成全市空间规划，形成城乡一体、全域管控的规划体系，优化生产、生活、生态空间布局，实现经济发展与生态保护的内在统一、相互促进。建立公众参与机制，通过立法确定规划的法律地位，切实增强规划的科学性、法定性和强制性。二是坚持产城融合。完成旧城改造工程，消除城市棚户区，加强历史文化遗迹保护，彰显城市文化底蕴，再现丝路古城风貌。实施清水河市区段综合治理、沈家河湿地保护等项目，建成黄土高原干旱半干旱地区海绵城市建设示范区、生态园林和文化旅游城市。实施西吉生态休闲公园、隆德产业园功能提升、泾源卧龙山森林公园二期、彭阳绿都颐养城等县城扩容提质工程。沿国省干线、旅游环线打造黄铎堡、将台、凤岭、兴盛、红河等10个特色镇和峡口、党家岔、联合、杨岭、高庄等100个美丽村庄。发挥特色街区、工业园区、五河流域城镇资源优势，积极发展农产品精深加工、商贸物流、连锁经营等业态，实现城区、园区同步发展。到2021年，全市户籍人口城镇化率提高到45%(常住人口城镇化率55%)，建成国家新型城镇化综合试点城市。三是精细管理城市。以建设“智慧固原”为抓手，整合信息网络资源，建设管理运行平台，加快推广智慧城管、智慧社保、智慧医疗、智慧教育等民生服务应用，构建一体化在线公共服务体系，实现城市管理信息化、基础设施智能化、惠民服务便捷化和社会治理精细化。健全保洁机制，提升管理水平，城市道路机械化清扫率达到100%。积极争创国家园林城市、卫生城市、水生态文明城市、森林城市和双拥模范城市。

　　三、倡导绿色，厚植绿水青山生态优势。坚持把生态作为最宝贵的优势，建好生态、护好生态、用好生态。一是践行绿色理念。划定生态红线，引导和约束开发行为，发展绿色产业体系。倡导绿色消费，推广绿色建筑，大力发展循环经济。强化全民节水意识，实行最严格的水资源管理制度和阶梯水价，加强中南部城乡饮水安全工程运行管理，加快节水型社会建设。二是织牢绿色屏障。围绕建设六盘山重点生态功能区，打造西部重要生态屏障，坚持不懈开展全民绿化行动，实施天然草原与湿地保护、荒山造林、绿色廊道、移民迁出区生态修复等重点工程，年降雨量400毫米以上宜林荒山荒地全部造林绿化，新增营造林260万亩以上。到2021年，森林覆盖率突破30%，水土流失治理程度达到95%以上。三是护好绿色家园。开展蓝天碧水绿色城乡专项行动，采用PPP模式引进大型企业实施五河流域综合治理，完成县城污水处理厂一级A提标改造，做到工业园区废水零排放;推进大气污染治理联防联控，彻底淘汰燃煤茶浴炉，加大对重点企业的监管，加强城市建筑施工扬尘、机动车尾气污染防治;深入推进农村环境综合整治，建设农村垃圾污水处理设施，实现五河流域出境断面水质稳定达标，年度环境空气质量优良天数达到90%，让良好的生态环境成为最公平的公共产品。四是发挥绿色优势。争取建立六盘山跨省生态补偿机制，加强水源涵养保护，让生态和水资源供给区得到补偿。实施六盘山集中连片特困地区水资源高效利用等工程，加快中型水库提升改造，高标准建成60万亩高效农业节水基地;建成引黄入固工程，到2021年，全市年供水能力达到2亿立方米。做强做优生态农业、生态旅游、健康养老等产业，把绿水青山变成金山银山，真正成为百姓的“摇钱树”。

　　四、改革开放，拓展跨越发展合作领域。以“敢开放、真开放”的姿态，构建进出畅通、内外一体的开放局面。一是优化发展环境。围绕改革主体框架，加快农村土地及林权、城镇化、社会保障、文化、金融、财政、社会管理等重点领域和关键环节改革，破障碍、出经验。推动“放管服”改革向纵深发展，全面实行网上办理，强化事中事后监管，进一步优化政务、政策、法治、市场、社会和人文环境，不断激发市场主体竞相发展的活力。适应新的投融资政策，通过九龙、交通、旅游、农投4家企业撬动更多社会资本、民间资金建设重大项目。二是建设开放通道。打通连接庆阳、宝鸡、兰州等高速断头路，高标准改造提升普通国省道612公里、农村公路2000公里。争取尽早开工宝中铁路扩能改造和固原火车站改扩建项目，实现高铁连通西兰银省会城市。改扩建六盘山机场，加密、开通更多航线，形成对外开放的空地快速通道。建成“宽带中国”示范城市，搭上网上丝绸之路快车。三是搭建合作平台。加强与周边城市在商贸物流、文化旅游、交通建设等领域的对接协作，扩大区位优势。深入落实“银川会议”精神，使闽宁对口帮扶更具实效。利用中阿博览会等平台，抢抓全国工商联宁夏创新发展大会机遇，主打“六盘山生态农产品”“六盘山生态旅游”两大品牌，开展产业精准招商、园区特色招商，五年引进资金1000亿元以上。

　　五、共建共享，满足人民群众美好愿景。坚持以人民为中心的发展思想，把财政支出更多用于提升人的素质能力、健康水平、公共服务等领域，促进群众获得感、幸福感与经济发展相协调。一是精准脱贫共享小康。把脱贫作为最根本的任务，完成“十三五”易地扶贫搬迁;实施“5·30”及其倍增计划和劳动力素质提升、两个带头人、金融扶贫等工程。实施全民参保计划，完善社会救助体系，兜住贫困群众生活底线。脱贫退出后政策不变、力度不减、队伍不散，确保2020年前贫困人口稳定脱贫、五县(区)全部摘帽。二是改善民生共享发展。促进教育资源公平普惠，完成义务教育均衡发展目标，普及高中阶段教育，实现学前教育城乡全覆盖，建成现代职业教育体系，坚决补齐学前教育和职业教育两个短板。把更多的资金投入到培养六盘名师、提高教师能力、保障教师待遇方面，建立一支人民满意的师资队伍。提供高效便捷的医疗服务，完成公立医院综合医改，实现公立医院在现有等级上再上水平，形成小病在基层院、大病在等级院、康复在社区中心的治疗格局，让群众看病更方便更满意。创新办医机制，加强与宣武、西京、宁医总院等知名医院合作，采取培养人才、远程会诊、专家来固就诊等方式提高医疗服务水平，让老百姓看得好病，切实减轻家庭负担，防止因病返贫。改革医院人事管理和收入分配制度，激励医技人员钻研技术，增长技能，改善服务，优劳多得，弘扬“治病救人、救死扶伤”医德，彰显“白衣天使”伟大形象。改进药招“三统一”方式，斩断利益链。通过化解医院债务、补贴费用支出等措施，大幅降低医院负担和老百姓看病成本。支持办好中医药事业和民营医院发展。推进健康固原建设，发展卫生与健康事业。鼓励和支持社会力量办养老事业。坚持计划生育基本国策，促进人口均衡发展。发展丰富多彩的文化事业，实施文化惠民工程，行政村综合文化活动中心全覆盖，做靓农村文化大院品牌。建设一支优秀文化队伍，支持创排反映脱贫攻坚、民族团结等方面的优秀文学艺术作品，做强文学固原品牌。加强文化遗产保护与传承，建成秦长城遗址公园，争取丝绸之路(宁夏固原段)申遗成功。创造更多创业就业机会，认真落实加快城乡居民增收意见，多渠道增加农民收入，破解农民增收“天花板”，确保农民收入增幅高于城镇居民。创建区级“双创”示范基地、创业就业孵化示范基地(园区)10个，每年新增城镇就业1万人以上。完善最低工资标准调整机制，提高城镇中低收入居民收入，让老百姓的“钱袋子”鼓起来。三是强化治理共享平安。加强民风建设，开展移风易俗宣传教育，促进群众转变观念、节俭办事;引导群众断掉“靠穷富”的念头，增强“幸福不会从天降”的意识，用勤劳的双手创造美好生活。深化“平安固原”创建，全面推进“网上信访”，把矛盾纠纷解决在基层和萌芽状态;推进社会治安防控体系建设，提升群众安全感。扎实开展“七五”普法，增强全民尊法学法守法用法意识。支持司法体制改革，保证司法公正，为经济发展、社会和谐提供坚强保障。开展民族团结“586”创建活动，打造“六盘儿女一家亲”品牌，落实“5+6+1”制度，不断提高宗教事务管理法治化、规范化水平，创建全国民族团结进步模范市。全方位加强安全生产，全过程强化食品药品监管，确保群众生命财产和饮食用药安全。

　　各位代表，实现上述目标任务，我们必须增强政治意识、大局意识、核心意识和看齐意识，按照“实干、团结、创新、高效、清廉”要求，建设人民满意的政府。一要勤学善谋强素质。深入学习贯彻习近平总书记系列重要讲话精神，牢固树立战略思维、系统思维、创新思维、长远思维;注重向群众学、向实践学，切实增强为民服务的公仆情怀;坚持“走出去”“请进来”，不断提高抢抓机遇、谋划工作、解决问题的能力，有效解决思想解放程度、谋划工作深度、贯彻落实精度、推进工作力度“四个不足”问题。二要敢于担当抓落实。牢固树立“权力就是责任、责任就要担当”的观念，坚持实干、苦干、巧干，深入基层，主动作为，真诚服务，做到守土有责、守土负责、守土尽责;强化效能问责，坚决整治庸政怠政懒政行为。三要依法履职严用权。全面推进依法行政，坚决拥护市委决定，主动接受市人大及其常委会的法律监督、工作监督和市政协的民主监督，广泛听取各民主党派和社会各界人士的意见建议;进一步推进政务村务公开，建立健全网络公开平台，推进决策、执行、管理、服务和结果“五公开”;自觉运用法治思维和法治方式推动工作，实现政府活动全面纳入法治轨道。四要廉洁从政树形象。坚守纪律规矩红线，严格执行党内政治生活若干准则和党内监督条例，不折不扣落实中央“八项规定”和有关规定，坚决践行新一届市委向全市人民作出的八条郑重承诺。扎实推进党风廉洁建设，加强反腐倡廉教育;全面推行涉农惠农项目资金“三级备案、三级审核、一个平台监管”工作机制，强化政府投资项目、扶贫资金等重点领域的行政监察和审计监督，从严查处“四风”问题和违纪违法案件，做到敬仰党的事业、敬畏手中的权力、敬重服务的人民。

　　2017年重点工作

　　全面贯彻落实党的十八届六中全会、中央经济工作会议和自治区党委十一届九次全会精神，准确把握稳中求进工作总基调，深入推进供给侧结构性改革，强化监测，综合施策，保持全市经济运行平稳、改革推进平稳、精准脱贫平稳、社会治理平稳，在基础性关键性领域实现新突破。按照市委四届一次全会部署，计划安排地区生产总值增长8%，全社会固定资产投资增长13%，地方公共财政一般预算收入可比增长10%，城乡居民人均可支配收入分别增长8%和9%，社会消费品零售总额增长8%。实现上述目标，全力抓好以下六项重点工作。

　　一、抓好精准脱贫帮扶。销号200个贫困村，减贫9.6万人，全市贫困发生率下降到7%以内。隆德县、泾源县、彭阳县脱贫摘帽，贫困发生率下降到3%以内;原州区、西吉县贫困发生率下降到8%以内。认真做好第三方考核评估工作，切实尽好帮扶责任。干好整村推进项目，整合投入25亿元，新改建农村公路62条500公里，农村自来水入户率达到85%。结合新型城镇化、美丽村庄建设，采取回购城镇住房、插花安置、就近建设安置点等方式易地搬迁安置2.1万人。加大对“两个带头人”的科技、项目支持力度，推进致富带头人提质扩量。通过土地流转盘活闲置土地、引进项目获取税收返还、加大村级财政转移支付等渠道增加村集体收入，每个县(区)发展2个示范村。增加农民工资收入，开展“冬季大培训、提振精气神”活动，精准培训5万人，就业率达到80%以上，3万人取得国家职业资格证书，就业率达到90%以上，保证贫困家庭至少有1人取得职业资格证书、掌握一门致富技能。转移农村劳动力30万人以上、创收60亿元以上。继续实施“5·30”及其倍增计划，做实做强到户脱贫产业，确保建档立卡贫困户收入增速高于农村居民。扩大金融扶贫范围，整合扶贫产业担保和风险补偿基金12亿元，探索建立商业保险担保机制，撬动银行贷款80亿元，实现有发展能力的建档立卡贫困户和脱贫销号户贷款全覆盖，贷款需求全满足。

　　二、抓好特色产业升级。重点推进五河流域产业带建设。做优生态农业，实施国家农业科技园区一期工程，建成园区科技创新服务中心、蔬菜育苗中心及道路、水利等配套设施，同步推进四县农业科技示范园建设。草畜产业突出抓点示范、全产业链建设，提高基础母牛“见犊补母”补助标准，新建标准化养殖园区5个、示范乡镇5个、示范村10个，肉牛饲养量达到111万头，其中安格斯肉牛4万头。启动建设全国高端肉牛繁育基地。林业和林下经济突出因地制宜、规模发展，建设油用牡丹基地3万亩、万寿菊基地10万亩，种植中药材3.5万亩，发展特色经果林1.45万亩、中蜂5000群、珍珠鸡等林下养殖78万只。马铃薯产业加强良种扩繁体系建设，生产脱毒原原种1.5亿粒，创建万亩马铃薯原种、一级种薯标准化生产基地10个26万亩，主食化品种面积2万亩;扶持国圣、勇兴等企业开展主食化产品研发，马铃薯淀粉加工能力达到15万吨。冷凉蔬菜产业创建标准化示范基地10个，生产面积达到61万亩。开拓西安、武汉等区外销售市场，创建外埠六盘山生态农产品营销网点5个。建成固原公益性农产品批发市场。引进培育带动能力强的龙头企业2-3家，新培育专业大户200户，规范发展家庭农场30家、农民合作社100家。提升全域旅游，建成固原至张易、火石寨至西吉等4条旅游环线和黄铎堡至须弥山等12条旅游精品线路。抓好原州区须弥山沿途景观、中庄和沈家河水库旅游景区、彭阳县茹河瀑布风景区、梯田公园建设等项目，提升改造西吉将台堡、震湖湿地保护、隆德老巷子、泾源二龙河、六盘山长征景区，高标准建设六盘山国际滑雪场并投入运营。大力发展乡村旅游，建成杨岭等乡村旅游示范村10个、六盘山民宿示范户200户。引进1家有实力的大企业开发固原旅游。全年接待游客500万人次，旅游社会总收入突破30亿元。壮大新型工业，出台加快经济技术开发区发展意见，完善开发区“一区三园”基础设施配套，引进入园企业20家，新增工业产值5亿元。金昱元盐化工循环经济产业链一期建成投产，支持发展下游产品项目。加快推进煤电铝一体化项目工作进程。全市完成工业总投资85亿元，增长20%;新增规上工业企业10家以上，规上工业增加值增长8%。

　　三、抓好重点项目建设。实施精准扶贫、旧城改造、五河流域发展、全域旅游、交通、美丽固原、工业、社会事业8大工程包63个项目，计划总投资1302亿元，年度计划投资357亿元，其中续建项目16个72亿元，新建项目47个285亿元。安排项目前期工作经费5000万元，继续开展“准备之冬、开工之春”活动，市、县(区)政府投资新建项目3月底前全部开工，企业投资项目6月底前全部开工。安排资金5000万元，重点支持国家农业科技园区、将台特色旅游小镇、隆德县城乡供水工程、杨岭美丽村庄、茹河瀑布风景区等8个市县共建项目，争取把五河流域综合治理、固原一中初中部扩建等项目列入自治区60周年大庆献礼项目库得到支持。建立驻京争项目争政策工作长效机制，争取国家专项建设资金(基金)50亿元，撬动民间投资200亿元以上。提升城乡改造质量，建成博物馆、城隍庙、西湖路等4个片区100万平方米棚改工程，博物馆文化产业城9月份投运。投资50亿元，加快清水河两岸、文化巷等片区1万户150万平方米棚改进程。完成文化路、政府路、南城路、中山街两侧建筑物立面改造和北京路、上海路街景风貌改造。基本建成古城墙遗址公园，完成清水河市区段综合治理项目。改造海绵化市区道路16条18公里、海绵型建筑小区90个323万平方米，完成120个海绵城市建设项目总工程量的80%，确保大部分城市建设工程明年基本完工，实现国家园林城市创建目标。继续实施县城棚户区改造，建成西吉县城集中供热改造、隆德“三馆”、泾源盈德生态旅游文化城、彭阳茹河街道路及供排水等县城建设项目，提升综合服务功能。集中建设张易、震湖、神林、大湾、城阳等5个特色乡镇和石羊子、西坪、联合、杨岭、高庄等15个美丽村庄。加速交通项目建设，开展交通建设攻坚年活动，加快建设S25线泾源至华亭、S60线西吉至会宁省际高速公路，完成投资16亿元;抓好G327线彭阳至沟圈等6条国省干线公路改造，完成投资10亿元;争取开工建设G85线银川至昆明高速公路固原段和宝中铁路扩能改造项目。完成固原火车站和六盘山机场改扩建等项目前期工作。提升水利保障能力，出台中南部城乡饮水安全工程运行管理办法，提高使用效益。实施六盘山集中连片特困地区水资源高效利用项目，发展节水灌溉面积5万亩，累计达到50万亩;新建乔家沟等5座水库，完成沈家河等6座水库清淤治污。建成隆德县城乡供水工程，力争开工建设引黄入固一期工程。提速信息服务水平，建成智慧城市展示中心、市级数据交换共享平台和政府综合服务平台;行政村光纤宽带全覆盖。建成“京东—宁夏扶贫特产馆”，培育电商龙头企业5家、扶贫示范村5个，地方特色农产品网络销售额突破1亿元。

　　四、抓好生态环境保护。安排1000万元先行建立市域内生态补偿机制。整合资金8.5亿元，实施六盘山重点生态功能区400毫米降水线以上区域造林绿化工程，新增营造林29万亩、补植补造13万亩。新建3000亩以上集中连片绿化点5个，完成城乡大绿化2万亩，抓好东岳山、北山、龟山、泾河源、茹河等城市休闲森林公园提升，全市森林覆盖率达到25.1%。治理小流域300平方公里。加大环境治理力度，完成清水河、葫芦河、渝河、泾河、茹河河道综合治理，淘汰万吨以下马铃薯淀粉加工企业，生产企业废水达标排放;提标改造市第一、第二和四县县城污水处理厂，新建新材料产业园和三营、兴隆等重点乡镇污水处理厂6座，全面取缔工业和生活污水入河入库直排口，泾河出境断面水质稳定在Ⅱ类以上，清水河、葫芦河、渝河、茹河出境断面水质稳定在Ⅳ类以上。淘汰全部黄标车和20蒸吨以下燃煤锅炉，通过湿法清扫、喷雾降尘等措施治理建筑工地扬尘，扭转PM10不降反升局面，空气质量优良天数达到90%。加强农村环境综合整治，农田残膜回收率达到86%以上。

　　五、抓好民生实事办理。统筹投入5亿元，办好自治区民生计划和我市13件民生实事(见附件)。加快义务教育均衡发展，西吉县通过自治区评估验收，原州区、隆德县、泾源县、彭阳县通过国家审核认定。加强职业教育和学前教育，实现市内中职教育一二年级学生免费、建档立卡贫困户适龄幼儿学前教育“一免一补”、行政村学前教育全覆盖。投资10.2亿元，新改建中小学校舍3.65万平方米、运动场7.9万平方米，新改扩建幼儿园130所。完成固原八中、市民族职业技术学院改扩建等重点工程。深入推进综合医改，实行分级诊疗，县域内就诊率达到90%;改革编制管理，打破身份界限，逐步实现同工同酬;改革医保支付方式，居民实际报销和大病报销支付比例分别提高到75%和50%以上。完成市区医疗资源整合，开展市区医院上等级创建活动。切实抓好卫生计生工作，助力脱贫攻坚。深入实施文化惠民工程，建成387个行政村综合文化服务中心，扶持发展20家市级示范性农村文化大院。实施秦长城遗址公园项目，争取开工建设固原博物馆改扩建项目，启动隋唐墓地遗址文化园建设。对建档立卡贫困户中符合农村低保条件的全部纳入低保，“两无人员”全部纳入低保政策兜底。建成市老干部活动中心(老年大学)。加快立体化社会治安防控体系建设，积极争取国家“雪亮工程”试点城市项目。创新建设模式和政府购买服务方式，加强安全监管信息化和应急救援能力建设;加大执法监管力度，坚决遏制重特大安全生产事故发生。深入开展矛盾纠纷排查调处和信访突出问题化解，依法严厉打击非法集资等各类犯罪活动，营造和谐稳定的社会环境。深入开展“两创建、两争做”“民族团结月”等活动，加强宗教教职人员培训，依法管理宗教事务和活动。推进民风建设，以“五美乡村”为载体，突出“一约四会”，遏制大操大办婚丧嫁娶、高额彩礼等陋习，切实减轻群众负担。

　　六、抓好重点领域改革。完成30项重点改革任务。五县(区)同步实施行政审批相对集中权改革，再造行政审批流程，加大权责清单动态监管。完成“多规合一”试点。推进城市执法体制改革，组建原州区综合执法局。县(区)成立农村产权流转交易中心(市场)，开展农村集体资产股份权能改革。完善四家集团公司资本运作机制，发挥统筹要素、配置资源、引领发展的主力作用。深化闽宁对口和区域协作，加强全国工商联常委会招商引资项目的对接洽谈，落实市级领导牵头责任和县区、部门分工任务，开展“民企固原行”“千企帮千村”行动，超额完成500亿元招商引资任务，招商引资到位资金增长15%。

　　面对新形势新任务，我们将忠诚履职，务实创新，勇于担当，廉洁用权，确保市委各项决策部署不折不扣落到实处，干出在全区有影响、有特色的亮点工作。

　　各位代表，攻坚拔寨一条心，众人能移万座山。让我们紧密团结在以习近平同志为核心的党中央周围，在自治区党委、政府和市委的坚强领导下，立愚公之志，聚全民之力，加快建设固原，实现经济繁荣、民族团结、环境优美、人民富裕，为打赢脱贫攻坚战、全面建成小康社会而努力奋斗!
